# Defective endoplasmic reticulum-mitochondria contacts and bioenergetics in SEPN1-related myopathy

**DOI:** 10.1038/s41418-020-0587-z

**Published:** 2020-07-13

**Authors:** Anne Filipe, Alexander Chernorudskiy, Sandrine Arbogast, Ersilia Varone, Rocío-Nur Villar-Quiles, Diego Pozzer, Maryline Moulin, Stefano Fumagalli, Eva Cabet, Swati Dudhal, Maria-Grazia De Simoni, Raphaël Denis, Nathalie Vadrot, Corinne Dill, Matteo Giovarelli, Luke Szweda, Clara De Palma, Paolo Pinton, Carlotta Giorgi, Carlo Viscomi, Emilio Clementi, Sonia Missiroli, Simona Boncompagni, Ester Zito, Ana Ferreiro

**Affiliations:** 1 Basic and Translational Myology laboratory, Université de Paris BFA, UMR 8251, CNRS, F-75013 Paris, France; 2 Istituto di Ricerche Farmacologiche Mario Negri - IRCCS, Milano, Italy; 3 Department of Biomedical and Clinical Sciences, “Luigi Sacco” University Hospital, Unit of Clinical Pharmacology, Università di Milano, Milano, Italy; 4 Division of Cardiology, University of Texas Southwestern Medical Center, Dallas, TX USA; 5 Department of Medical Sciences, Laboratory for Technologies of Advanced Therapies (LTTA), University of Ferrara, Ferrara, Italy; 6 Department of Biomedical Sciences, University of Padova, Padova, Italy; 7 CeSI-Met, Centro Scienze dell’Invecchiamento e Medicina Traslazionale & DNICS, Department of Neuroscience, Imaging and Clinical Sciences, University G. d’Annunzio of Chieti, Chieti, Italy; 8 Reference Center for Neuromuscular Disorders Nord/Est/Ile-de-France, APHP, Institut of Myology, Pitié-Salpêtrière Hospital, Paris, France; 9 Present Address: PhyMedExp, University of Montpellier, INSERM, CNRS, Montpellier, France

**Keywords:** Chaperones, Respiratory tract diseases

## Abstract

SEPN1-related myopathy (SEPN1-RM) is a muscle disorder due to mutations of the *SEPN1* gene, which is characterized by muscle weakness and fatigue leading to scoliosis and life-threatening respiratory failure. Core lesions, focal areas of mitochondria depletion in skeletal muscle fibers, are the most common histopathological lesion. SEPN1-RM underlying mechanisms and the precise role of SEPN1 in muscle remained incompletely understood, hindering the development of biomarkers and therapies for this untreatable disease. To investigate the pathophysiological pathways in SEPN1-RM, we performed metabolic studies, calcium and ATP measurements, super-resolution and electron microscopy on in vivo and in vitro models of SEPN1 deficiency as well as muscle biopsies from SEPN1-RM patients. Mouse models of SEPN1 deficiency showed marked alterations in mitochondrial physiology and energy metabolism, suggesting that SEPN1 controls mitochondrial bioenergetics. Moreover, we found that SEPN1 was enriched at the mitochondria-associated membranes (MAM), and was needed for calcium transients between ER and mitochondria, as well as for the integrity of ER-mitochondria contacts. Consistently, loss of SEPN1 in patients was associated with alterations in body composition which correlated with the severity of muscle weakness, and with impaired ER-mitochondria contacts and low ATP levels. Our results indicate a role of SEPN1 as a novel MAM protein involved in mitochondrial bioenergetics. They also identify a systemic bioenergetic component in SEPN1-RM and establish mitochondria as a novel therapeutic target. This role of SEPN1 contributes to explain the fatigue and core lesions in skeletal muscle as well as the body composition abnormalities identified as part of the SEPN1-RM phenotype. Finally, these results point out to an unrecognized interplay between mitochondrial bioenergetics and ER homeostasis in skeletal muscle. They could therefore pave the way to the identification of biomarkers and therapeutic drugs for SEPN1-RM and for other disorders in which muscle ER-mitochondria cross-talk are impaired.

## Introduction

Endoplasmic reticulum (ER) and mitochondria are essential organelles which control multiple cellular processes. In response to metabolic and energy requirements, ER participates in glucose and lipid anabolism and protein synthesis, folding and sorting, while mitochondria produce most cellular ATP during glucose and lipid catabolism [1, 2]. The ER communicates with mitochondria through contact sites termed mitochondria-associated membranes (MAMs), which link both organelles through proteinaceous tethers whose molecular identity is only partially identified [3]. Dynamic adjustment of MAMs structure is an adaptive prerequisite for metabolic responses to nutritional conditions or environmental stimuli, while altered MAM contacts have been associated with pathology [4, 5]. Thus, changes in nutrient availability lead to MAM remodeling and consecutive adaptation of mitochondrial respiration [6]. Conversely, alterations of MAM integrity have been associated with metabolic dysfunctions such as defective glucose homeostasis and insulin sensitivity [3, 4, 7], and also with neurological disorders including amyotrophic lateral sclerosis, Parkinson’s disease, cerebral ischemia, or Huntington’s disease [8]. Identification of all the relevant MAM proteins is therefore important for understanding human neuromuscular pathophysiology and for identifying novel therapeutic targets and approaches.

SEPN1-related myopathy (SEPN1-RM) is an autosomal recessive congenital muscle disease due to inherited defects of Selenoprotein N, encoded by the *SELENON* (or *SEPN1*) gene. SEPN1-RM typically presents in infancy with severe neck and trunk muscle weakness which can lead to death due to respiratory failure [9]. Limb muscle weakness and fatigue are also present and, in severe forms of the disease and/or in late adulthood, can cause loss of ambulation [10]. Most SEPN1-RM muscle biopsies show small focal areas of mitochondrial depletion and sarcomere disorganization (minicores) in muscle fibers. Necrosis and regeneration and/or protein aggregates are less frequent but may also be present. This rare genetic disease has no treatment so far.

SEPN1 or SELENON is a ubiquitous endoplasmic/sarcoplasmic reticulum (ER/SR) protein [11] which protects cells against oxidative stress [12, 13] or ER-stress [14, 15] and defends calcium homeostasis by counteracting ERO1-mediated oxidation of the ATP-dependent Ca^2+^ pump SERCA [16, 17]. However, it is still unclear how the lack of functional SEPN1 leads to the phenotype in SEPN1-RM patients. One unexplained clinical feature is the fatigue which affects functionally key muscles such as the diaphragm [18] and has a major impact on patients’ functional and respiratory capacities and quality of life. Other open questions are the origin of the minicores in patients’ muscles, or the discordance between the severe weakness of neck and trunk muscles and the relatively preserved strength of limb muscles, causing major respiratory failure in ambulant patients. Extramuscular abnormalities such as paradoxical insulin resistance [15] or abnormal body mass regulation, with cachexia [13] and altered fatty tissue distribution [19, 20], have been observed in SEPN1-RM patients and remain incompletely understood.

Here, we show that SEPN1 is enriched at MAMs and its absence leads to reduced ER-mitochondria contacts, low organelle Ca^2+^ content, and impaired oxidative phosphorylation (OXPHOS). Our results indicate a new role of SEPN1 in the interplay between mitochondrial bioenergetics and SR homeostasis in skeletal muscle, identify a systemic metabolic/bioenergetic component in SEPN1-RM and suggest mitochondria as a novel therapeutic target.

## Materials and methods

### Animal model

The *Sepn1* KO mouse model has been described previously [21]. All studies followed the European guidelines for the care and use of laboratory animals in accordance with the 1964 Declaration of Helsinki and its later amendments, after approval by the Paris Diderot University/Université de Paris or Mario Negri Institute ethics committee. To minimize the number of animals used, we performed in vivo studies in female animals (*Sepn1* KO males having a comparable or more marked phenotype [21]) and confirmed the abnormalities ex vivo using muscles from males. Animals were allocated to experimental groups based on their genotype. Investigators were blinded to genotype during experiments and/or when assessing the outcome.

### Acute exercise study

We followed strictly the treadmill protocol in [22]. Exhaustion was defined as the point at which mice spent more than 5 s on the side of the lane.

### Blood glucose measurements

Glucose levels on tail-vein blood were analyzed using a commercial glucometer and strips (One Touch).

### In vivo metabolic cage studies

Mice were individually housed with water and food ad libitum (A030, 2.83 kcal/g, SAFE, France), lights on 7 a.m. to 7 p.m. and temperature of 22 ± 0.5 °C. Mouse weight and whole-body composition (fat mass and lean body mass (LBM)) were measured with an Echo Medical Systems EchoMRI 100 (Whole-Body Composition Analyzers, EchoMRI, Houston, TX). Total energy expenditure, oxygen consumption and CO_2_ production, respiratory exchange rate (RER, VCO_2_/VO_2_) and food intake (Labmaster; TSE Systems, Bad Homburg, Germany) were analyzed in calorimetric cages after 48 h acclimatation (Even and Nadkarni, 2012). FA oxidation rate was calculated as described [23]: FA oxidized (kcal/h) = EE (kcal/h) × (1 − RER/0.3).

### Ex vivo oxygen consumption in muscle fibers

Respiration was measured in permeabilized muscle fibers [24, 25]. Oxygen flux rates were expressed per tissue wet weight and per mtDNA copy number. Mitochondrial DNA (mtDNA) from muscle samples was quantified as described [25].

### PAS staining

Cryosections of gastrocnemii and liver were stained for Periodic acid-Schiff (PAS) following standard procedures [15], using amylase to confirm staining specificity.

### Quantification of activity of the Krebs cycle enzymes

Enzyme activities were assessed as in [26] using tibialis anterior muscles from WT and *Sepn1* KO mice.

### Plasmids, antibodies, and reagents

Plasmids expressing cMyc-tagged mouse SEPN1 (with A1284T/C conversion of the TGA selenocysteine codon into Cys to increase expression) and cMyc-tagged human Desmin were constructed in a CMV promoter-derived mammalian expression vector.

Expression plasmids encoding N-terminally FLAG-tagged human SEPN1 and the mutants SEPN1 SS (C427S, U428S) and SEPN1 CC (U428C) were constructed in pSel-Express vector (a kind gift from Vladimir Gladyshev) [16].

The primary antibodies used were: anti-VDAC2, anti-Cyt C, anti-Calnexin, anti-SERCA2, anti-RyR, anti-cMyc, and anti-PDI from Abcam; anti-Flag, anti-β-tubulin, and anti-Sigma 1-R from Sigma-Aldrich; anti-IP3R3 and Cyt c from BD Biosciences; anti-Tom40 from Santa Cruz, and homemade rabbit anti-SEPN1 [21]. Secondary antibodies Alexa Fluor 488 and 594 were from Life Technologies.

### Primary myoblasts, C2C12, and HeLa cells

Mouse satellite cells were extracted from muscles of 4-week-old wild-type (WT) mice, as described [27], maintained in proliferation medium 24 h post-transfection then in differentiation medium for 4 days. SEPN1 KO HeLa cells were generated with the CRISPR/CAS9 technology (OriGene) and have been previously described [14]. SEPN1 WT and KO C2C12 cells were obtained following adenoviral infection [15]. Four days post-infection quantitative PCR confirmed *Sepn1* deficiency (Primer Forward: GCTTTCCTGTAGAGATGATG, Primer Reverse: GCCCCGCCGGAGTCCTTC).

### MTS assay

Six thousand cells/well were plated in 96-well plates and treated as indicated in CellTiter 96^®^ Aqueous Non-Radioactive Cell Proliferation Assay (Promega).

### Subcellular fractionation

Fractionations from cells and tissues were performed as described [28, 29]. IP3R3 and PDI, Sigma 1-R, Tubulin, and Cytochrome c were used as markers for the ER (IP3R3 and PDI), MAMs (Sigma 1-R), cytosol (Tubulin), and pure mitochondria (Cytochrome c), respectively.

### Super-resolution microscopy

Structured illumination microscopy (SIM) was done on a Nikon SIM system with a 100 × 1.49 objective and images were assessed as in [15].

Briefly, randomly selected cells were imaged at laser excitation of 405 nm (nuclei), 488 nm (SIGMA 1-R-GFP), 561 nm (ER or SEPN1), and 640 nm (mitochondria) with a 3D-SIM acquisition protocol. SIM images were quantified with ImageJ. Co-localization between SIGMA 1-R-GFP (channel 1) and SEPN1 (channel 2) was analyzed using the Jacop ImageJ plugin and expressed as Manders 2 index. After background normalization, the ER and mitochondria signals were segmentated to calculate the area of the ER and that of the overlapping ER and mitochondria, both expressed as fraction of total cell area. The segmentated mitochondrial signal was followed by the skeletonize function of ImageJ to perform mitochondria network analysis. We then applied the Analyze Skeleton 2D/3D plugin of ImageJ to measure each cell mean branch length, expressed as μm.

### Measurement of calcium content and flux

HeLa cells grown on 13-mm-round glass coverslips were transfected with mitochondrial (mt) or ER targeted aequorin (Aeq) chimeras as described [30] together with SEPN1 WT, SEPN1 CC, SEPN1 SS, or empty vector. For the mtAeq experiments, cells were incubated with 5 μM coelenterazine for 2 h in 0.1% FBS medium, then transferred to the perfusion chamber as in [14]. Agonists were added to the medium as specified in the figure.

### ATP content

Cellular ATP content was determined using the ATP Determination kit following manufacturer’s instructions (Thermo Fisher Scientific) and normalized to protein concentration.

### Measurements of mitochondrial membrane potential

Mitochondrial membrane potential (Ψm) was assessed by loading cells with 10 nM tetramethyl rhodamine methyl ester (TMRM; Life Technologies, T-668) for 35 min at 37 °C in KRB supplemented with 1 mM CaCl_2_ as in [31].

### Biochemical analysis of MRC complexes

Samples were snap-frozen and homogenized in 10 mM phosphate buffer (pH 7.4). Spectrophotometric activity of CI, CII, CIII, and CIV was measured as described in [32].

### Real-time RT PCR

Total RNA was isolated from diaphragms using the RNeasy Mini Kit (Qiagen) in accordance with the manufacturer’s instructions. One microgram of total RNA was reverse-transcribed and analyzed using the Applied Biosystems’ real-time PCR System and the ΔΔCt method. Relative gene expression in muscle was normalized to *GAPDH* mRNA levels. Primers sequence was reported in [14].

### SEPN1-RM patient studies

We analyzed retrospectively anthropometric and clinical data from 27 patients after diagnostic identification of *SEPN1*/*SELENON* mutations using genomic DNA as described [10], after informed consent according to local ethical committees. Their global phenotype has been reported as part of a large case series [20].

Body mass index (BMI) was calculated as described in [33] for adults aged ≥ 20 years, and according to BMI-for-age percentile growth charts [34] in patients ≤20 years. Our criteria of disease severity were: (a) marked neonatal hypotonia; (b) scoliosis and/or respiratory failure before age 10 years; (c) progressive motor disability causing loss of ambulation before adulthood. Three severity groups were defined: severe (two or more criteria), moderate (one criterion), and mild (respiratory failure detected after the age of 20 years and/or, for younger patients, a clear objective trend to amelioration).

Primary human fibroblasts, amplified as described [11, 12], were obtained from skin biopsies after informed consent from three patients with the *SEPN1* mutations shown below and 4 age- and passage-paired controls.

**Table:** 

*SEPN1/SELENON* mutation	Gender	Age at biopsy	Passage used
c.1A>G (p.Met1Val) homozygous	M	16 years	P8-P10
c.713dupA(p.Asn238Lysfs*63)/c.1397G>A (p.Arg466Gln)	M	32 years	P9-P10
c.713dupA (p.Asn238Lysfs*63) homozygous	F	17 years	P10-P11

For electron microscopy, we used three available fixed and epoxy-embedded muscle samples from the third patient above and from two unrelated SEPN1-RM patients homozygous for the same mutation (c.713dupA, p.Asn238Lysfs*63), leading to protein absence by western blot. Biopsies were obtained for diagnosis after informed consent from deltoid (*N* = 2) or abdominal (*N* = 1) muscles at ages 8–12 years, and compared with deltoid from a 12-year-old control. Ultrathin sections (~40 nm) were cut in a Leica Ultracut R microtome (Leica Microsystem, Austria) and examined after double-staining with uranyl acetate and lead citrate on a FP 505 Morgagni Series 268D electron microscope (FEI Company, Brno, Czech Republic), with Megaview III digital camera (Munster, Germany) and Soft Imaging System (Germany). In each EM image, we determined the mitochondrial positioning with respect to the sarcomere A band [35] and the mitochondrial volume [36].

### Statistics

**Fig. 1:**
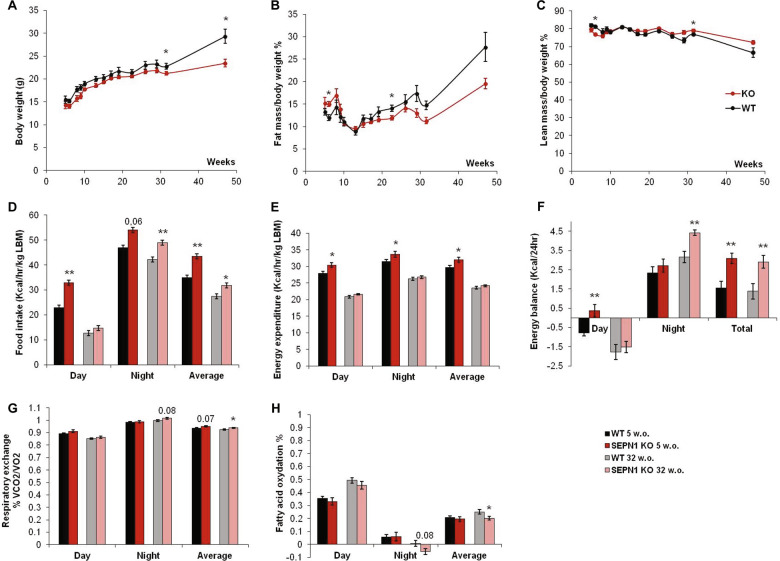
*Sepn1* KO mice present a metabolic phenotype in vivo with modified body mass composition, altered energy balance and defective fatty acid oxidation. Follow-up of body mass composition and calorimetric parameters between ages five (*N* = 12 mice per group) and 47 weeks (*N* = 6 mice per group). **a** Body weight; **b** percentage of fat mass and **c** lean mass over total body weight. Metabolic cage parameters during resting (day), activity (night), and average are shown: **d** food intake, **e** energy expenditure per kg of lean body mass, **f** energy balance corresponding to the difference between food intake and energy expenditure, **g** respiratory exchange ratio RER = 0.7 corresponding theoretically to 100% fatty acid oxidation, RER = 1 corresponding to 100% carbohydrate metabolism and **h** percentage of fatty acid oxidation in 5- and 32-week-old (w.o.) WT and SEPN1 KO.

**Fig. 2:**
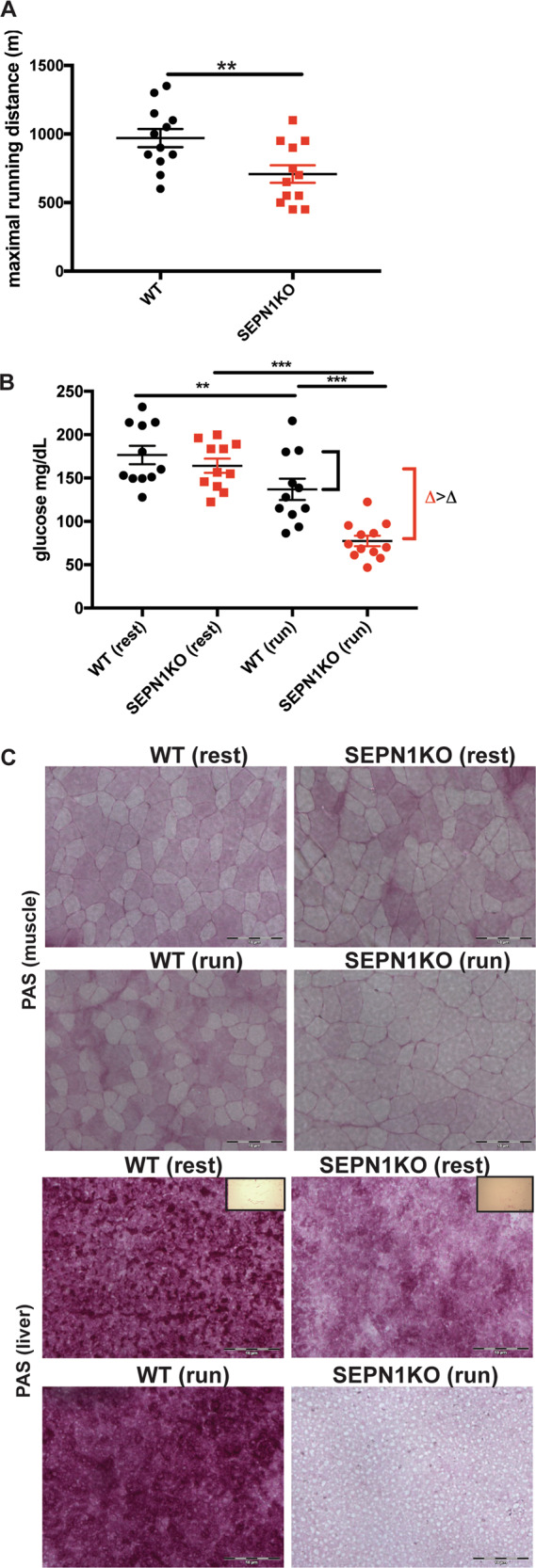
*Sepn1* KO mice show defective exercise endurance and increased muscle glucose metabolism during exercise. **a** Maximal treadmill running distance for 2-month-old mice of indicated genotype (*N* = 12). **b** Plasma glucose in mice of indicated genotype at rest and 20 min after exhaustive running was tested (*N* = 12). **c** Representative periodic acid-Schiff (PAS) staining in liver and gastrocnemius muscle sections from mice of indicated genotype, at rest and after running. Insets represent samples digested with amylase.

**Fig. 3:**
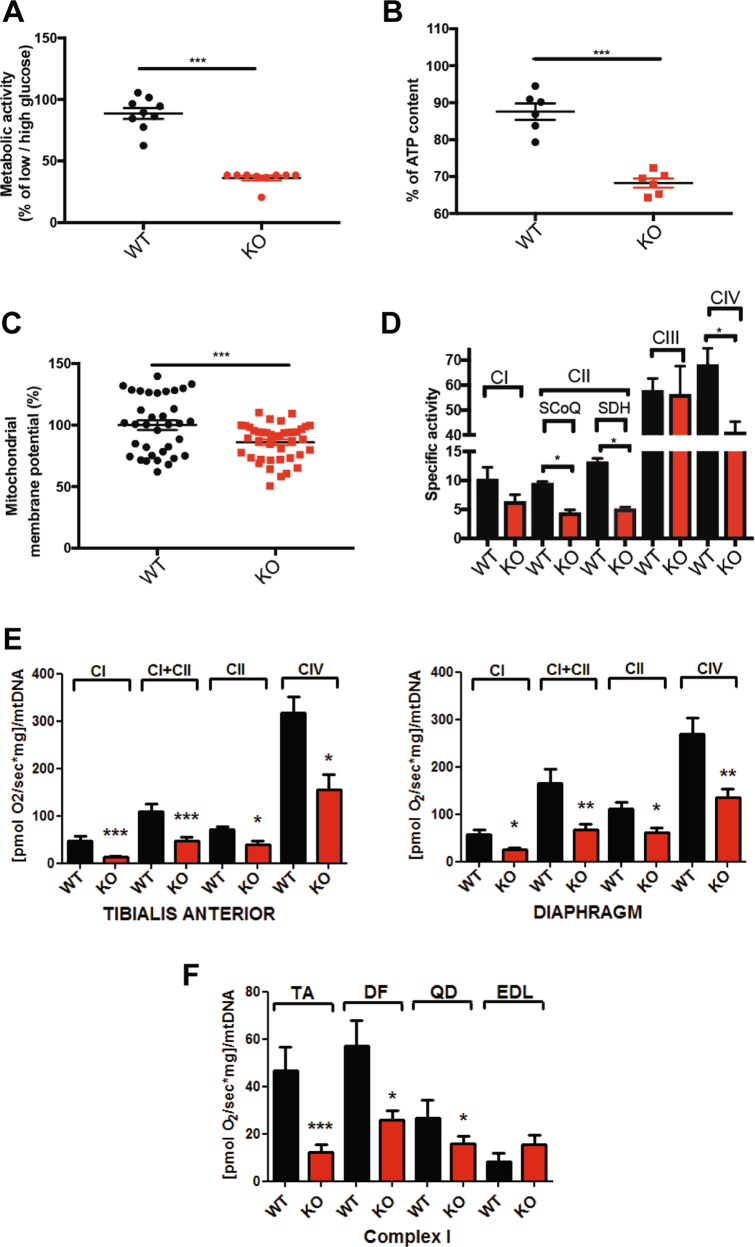
SEPN1 controls mitochondria bioenergetics. **a** Percentage of the ratio of the metabolic activity (MTS assay) of WT and SEPN1 KO HeLa cells between 2 days of culture in low glucose (5.5 mM) and high glucose (25 mM) (*N* = 9). **b** Measurement of cellular ATP content in WT and SEPN1 KO HeLa cells. Results from three independent experiments are summarized as a graph (*N* = 6). **c** Analysis of mitochondrial membrane potential (Ψm) as measured by TMRM intensity in WT and SEPN1 KO HeLa cells (*N* = 34). **d** Mitochondrial respiratory chain specific activity in WT and SEPN1 KO C2C12 (*N* = 4). **e** Oxygraphy studies of mitochondrial complexes in tibialis anterior and diaphragm muscles from *Sepn1* KO mice (red bars) compared with WT animals (black bars). **f** Complex I activity in tibialis anterior (TA), diaphragm (DF), quadriceps (QD), and extensor digitorum longus (EDL).

Data represent mean ± SEM unless otherwise indicated and were analyzed by Prism 6 (Graphpad) and IBM SPSS statistics 22.0. N as indicated in the figure legend except for dot plots. For two group analysis, unpaired *t* test (Figs. 1d–h, 2a, 3a–f, 5f–h, 7b) or nonparametric Mann–Whitney (Fig. 1a–c) were used. We used two-way ANOVA followed by Fisher’s test multiple comparison for Fig. 5b–d, and one-way ANOVA multiple comparison test for three or more group analysis (Figs. 2b, 6a–b). For phenotypical patient studies, contingency tables were used to compare relative frequencies in disease severity between groups, followed by Chi square test. One asterisk was used for *p* < 0.05, two for *p* < 0.01, three for *p* < 0.001 and four for *p* < 0.0001.

## Results

### *Sepn1* KO mice show defects in body composition, energy balance, and metabolic efficiency

To investigate whether the amyotrophy, weakness, and/or fatigue in SEPN1-RM patients are associated with a metabolic/bioenergetics defect, we performed in vivo metabolic studies of *Sepn1* KO mice compared with WT animals.

Body weight, lean, and fatty mass evolution were monitored by performing 13 scans (EchoMRI) for each mouse sequentially from the fifth to the forty seventh-week of age (Fig. 1a–c). Bioenergetics status was determined at the age of 5 weeks, immediately after weaning (basal, *N* = 12 per genotype), and at 32 weeks (*N* = 6 per group) (Fig. 1d–h).

Young postweaning *Sepn1* KO mice presented significant metabolic abnormalities, suggesting reduced bioenergetics efficiency (Fig. 1). These included significantly reduced lean mass, increased fat mass (expressed as percentage of total body weight), and global body weight that tended to be lower than in WT (Fig. 1a–c). Unexpectedly, KO mice also showed higher food intake (absolute and relative to LBM) and greater energy expenditure per LBM (Fig. 1d, e), associated with a significantly increased diurnal resting metabolism (data not shown) compared with WT. Consistently, although the cage energy balance was significantly more positive in KO animals (Fig. 1f), their body weight tended to stay lower and they did not gain more weight than WT during the 1-week metabolic cage study. Taken together, these data suggest energy dissipation for KO mice.

This profile was similar at 32 weeks but, consistently with observations in patients, some of the body composition abnormalities intensified with age. In adult *Sepn1* KO mice, body weight became more significantly reduced compared with WT, mainly due to lower fatty body mass from the age of 15 weeks, despite persistence of a higher food intake and a positive cage energy balance, particularly during the activity period (Fig. 1a–f). Energy expenditure and resting metabolism became comparable with WT, but adult *Sepn1* KO animals showed an elevated respiratory quotient suggesting an altered balance between fatty acid oxidation and carbohydrate metabolism in favor of the latter (Fig. 1g–h).

Altogether, these data identified for the first time an in vivo metabolic phenotype in a SEPN1-RM model, marked by lower body mass, increased energy expenditure and reduced bioenergetics efficiency, suggesting a novel role of SEPN1 in bioenergetics.

### Defective exercise endurance and increased muscle glucose metabolism in *Sepn1* KO mice

Next, we examined the consequences of SEPN1 absence on fatigue during strenuous exercise in 2-month-old mice. Strenuous exercise revealed 35% reduced exercise tolerance and fatigue in *Sepn1* KO mice compared with WT littermates (*N* = 12 per group) (Fig. 2a).

Previous gastrocnemii transcriptomic profiling and phenotype ontology analysis indicated a *Sepn1* KO muscle phenotype compatible with abnormal glucose metabolism [17]. Interestingly, *Sepn1* KO mice showed a tendency to lower blood glucose levels in basal conditions that was significant after exercise (Fig. 2b), suggesting that they depend on glucose more than controls during exercise. Consistently, although basal glycogen content was similar in WT and KO gastrocnemii, exercise-induced glycogen depletion was faster in muscle and also in liver (the main glycogen storage organ) of *Sepn1* KO mice (Fig. 2c).

Thus, *Sepn1* KO muscles show exercise intolerance and critically rely on glucose metabolism.

### SEPN1 depletion causes reduced activity of mitochondrial respiratory complexes

To investigate whether the phenotype described above was due to impaired mitochondrial function and compromised oxidative phosphorylation (OXPHOS), we developed two SEPN1-deficient cell models, in HeLa cells using CRISPR/CAS9 and in myogenic C2C12 (SEPN1 KO) through adenoviral-mediated shRNA. To determine whether SEPN1-devoid cells were more reliant on glucose metabolism, we cultured WT and SEPN1 KO cells for 2 days in high (4.5 g/L) or low glucose (1 g/L) and measured cellular metabolic rate by MTS assay. SEPN1-devoid cells were 50% less metabolically active in low glucose than controls, indicating that they are more dependent on glucose (Fig. 3a). Importantly, SEPN1-devoid cells had a 25% reduction in ATP content (Fig. 3b), a moderately lower mitochondrial membrane potential (Ψm) (Fig. 3c), reduced complex I activity and a significant defect of the OXPHOS complexes II and IV (Fig. 3d), suggesting a bioenergetics defect due to OXPHOS impairment.

Consistently, oxygraphy studies of saponine-permeabilized isolated muscle fiber bundles, randomly selected and obtained from different muscles of *Sepn1* KO and WT mice (*N* = 6–8 animals per group), showed that SEPN1 absence was associated with a dramatic reduction of mitochondrial respiration (Fig. 3e), more conspicuous in slow and mixed-fiber muscles (tibialis anterior, diaphragm, and quadriceps). Accordingly with preferential use of glycolysis, no difference in complex I activity was observed in the fast, glycolytic EDL muscle between *Sepn1* KO mice and WT (Fig. 3f). Reduced activity of mitochondrial complexes was not associated with significant changes in mitochondrial content or in expression levels of the master mitochondrial regulator PGC1α gene in *Sepn1* KO muscles compared with WT (data not shown).

To investigate whether SEPN1 regulates ATP production via the Krebs cycle, we quantified the activity of its key proteins in diaphragm muscles from *Sepn1* KO mice (*N* = 5, aged 2 months). SEPN1 absence led to a mild increase in pyruvate dehydrogenase activity (often nonspecifically associated with cellular oxidative stress), without significant differences in the activity of citrate synthase, aconitase, or KGDH between muscles from *Sepn1* KO mice and from WT littermates (data not shown), indicating a functional Krebs cycle in the former [37]. Altogether, these results suggest that SEPN1 may be a novel regulator of OXPHOS and mitochondrial ATP production in skeletal muscle.

### SEPN1 is enriched at the MAMs and controls ER-mitochondria calcium transfer

**Fig. 4:**
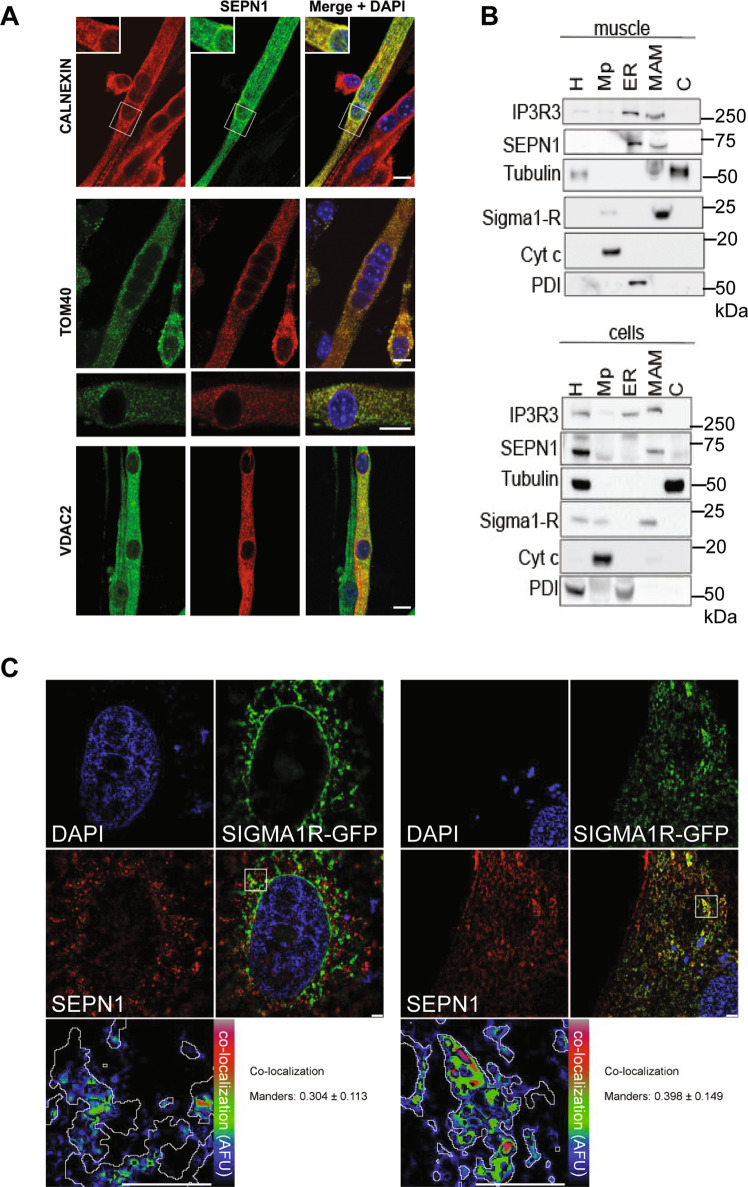
SEPN1 is a MAM-localized protein in nonmuscular and muscular cells. **a** Localization of a cMyc-SEPN1 in murine primary myotubes. Skeletal muscle cells were transfected with a plasmid expressing a cMyc-SEPN1 protein, which co-localized with calnexin (an ER marker), with the outer mitochondrial membrane protein TOM40 and with VDAC2 (a MAM marker). Scale bar is 10 µm. **b** Protein components of subcellular fractions prepared from muscle (gastrocnemius) and cells (HeLa) revealed by western blot analysis. SEPN1 presence was shown using a specific antibody. IP3R3 and PDI were used as ER markers, β-tubulin as a cytosolic marker, Sigma 1-R as a MAM marker, cytochrome C (Cyt c) as a mitochondrial marker. H homogenate, Mp pure mitochondria, ER endoplasmic reticulum, MAM mitochondria-associated membranes, C cytosol. **c** Co-localization of SEPN1-Flag (red) and Sigma 1-R-EGFP (a MAM marker, green) in C2C12 (right panel) and HeLa cells (left panel). The lower-right panel displays the merged image of the two stains. The lower-left panels display the SEPN1 signal overlaid with MAMs (MAM boundaries are highlighted in gray) in a rainbow lookup table (LUT). MAMs-SEPN1: Manders coefficient for SEPN1 staining was calculated as the proportion of SEPN1 signal overlapping with the Sigma 1-R marker.

No antibody recognizes specifically the endogenous SEPN1 on immunocytochemistry. Thus, to analyze whether the bioenergetics role of SEPN1 in muscle cells involves an intrinsic mitochondrial localization, we generated myotubes from primary murine satellite cells expressing exogenous SEPN1 tagged with cMyc. SEPN1 co-localized with the SR proteins calnexin (Fig. 4a), RyR, and SERCA2 (data not shown). We found no consistent co-localization of cMyc-SEPN1 with the mitochondrial matrix protein ATP5B (data not shown). However, there was a clear and intense co-localization of SEPN1 with the outer mitochondrial membrane proteins TOM40 and VDAC2 (Fig. 4a). Moreover, cell fractionation studies of the endogenous SEPN1, further substantiated by co-localization studies of FLAG-tagged SEPN1 with Sigma 1-R (a MAM marker), showed that SEPN1 was enriched at the MAMs [3, 38] (Fig. 4b, c), both in myogenic and in non-myogenic cells.

**Fig. 5:**
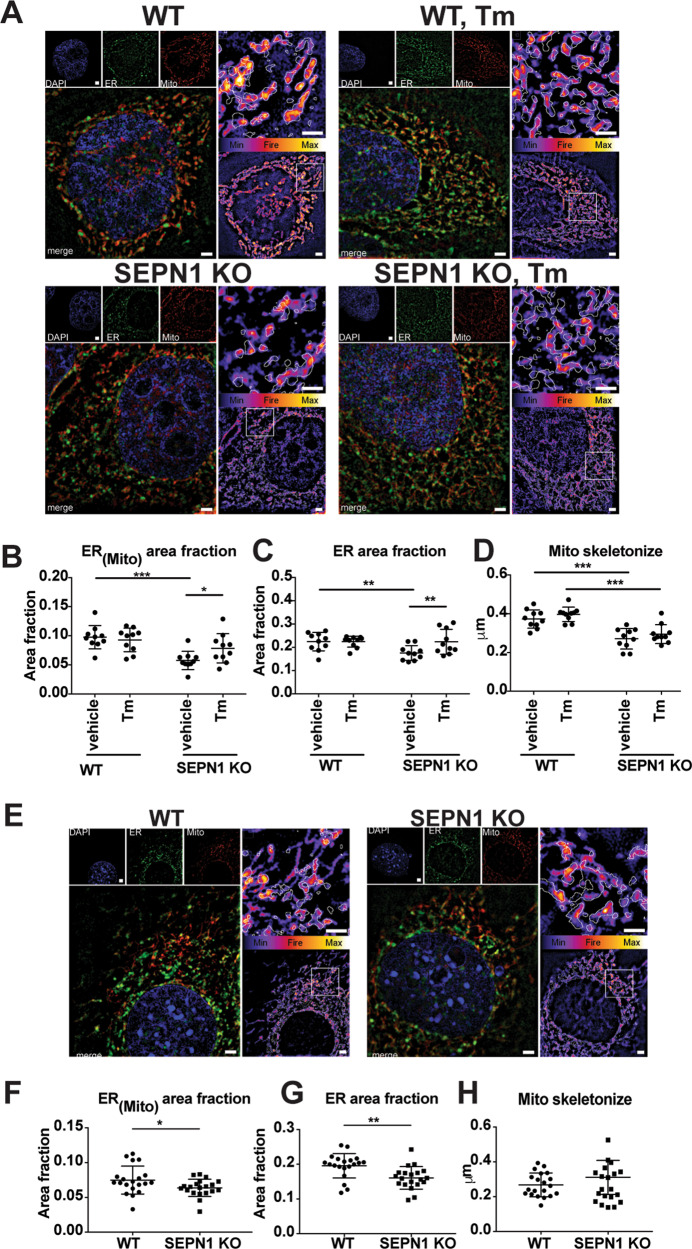
SEPN1-devoid cells display less ER-mitochondria apposition (MAMs). **a** Representative images of ER (green) and Mito (red) trackers in WT and SEPN1 KO HeLa cells following 16 h of tunicamycin (1 μg/mL) or vehicle. The outline of the ER tracker signal was used to create a mask within the Mito stained area that was quantified. The signal of Mito is shown as a color-coded fire image overlaid by the ER mask. **b** Dot plots representing the Mito area fraction in the ER mask decreased in SEPN1 KO HeLa cells (ER_(mito)_ area fraction). **c** The size of ER (ER area fraction) in HeLa cells. **d** Mitochondrial skeletonized signal was used to evaluate the mitochondria network. The mean branch length was calculated in WT and SEPN1 KO HeLa cells. **e** Representative images of ER (green) and Mito (red) trackers in WT and SEPN1 KO C2C12 cells. **f** The Mito area fraction in the ER mask decreased in SEPN1 KO C2C12 cells (ER_(mito)_ area fraction). **g** The size of ER (ER area fraction) in C2C12 cells. **h** Mitochondrial skeletonized signal was used to evaluate the mitochondria network. The mean branch length was calculated in WT and SEPN1 KO C2C12 cells. Each dot represents one cell.

To investigate ER, mitochondria and MAMs in a SEPN1-deficient cell context, we used a combination of endoplasmic reticulum- and mitochondria-specific trackers together with super-resolution microscopy. A higher degree of MAMs was observed in SEPN1 KO cells treated with the MAM-inducer tunicamycin [39] compared with untreated cells (Fig. 5a–d), validating our MAM-detection method. Interestingly, shrinkage of the overlapping region between the ER and mitochondria indicated fewer MAMs in SEPN1 KO cells versus WT (HeLa and C2C12) (Fig. 5a, b, e, f) together with a decrease in ER area fraction (Fig. 5c, g). To analyze if there was any difference in the mitochondria network, we skeletonized the signal associated with mitotracker as previously described [15]. We calculated the mean branch length (the segment connecting node-to-node or node-to-end point) in each cell as an index of the mitochondria network. In SEPN1 KO HeLa cells, the mitochondria network was impaired when compared with the WT counterpart (Fig. 5d) whereas no significant difference was seen in SEPN1 KO C2C12 cells (Fig. 5h). In addition, upregulation of CHOP, ERO1 and GADD34 expression in diaphragm muscles of old SEPN1 KO mice confirmed the existence of overt ER stress/maladaptive ER stress response in SEPN1-devoid muscles, consistently with our previously reported results [14, 15, 17] (Supplementary Fig. 1).

**Fig. 6:**
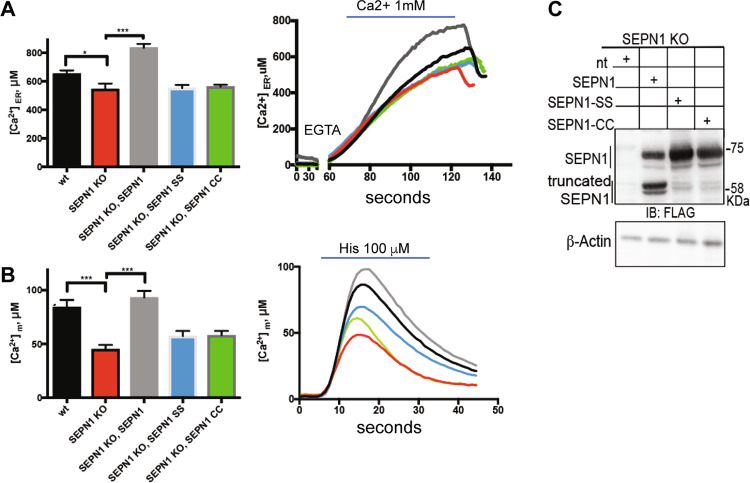
SEPN1 regulates ER-mitochondria Ca^2+^ homeostasis. **a** WT and SEPN1 KO HeLa cells were co-transfected with ER aequorin-based Ca^2+^ sensors and SEPN1 plasmids and calcium refilling of the ER was recorded (Ca^2+^ 1 mM) (*N* = 10). The traces are representative of ten independent experiments that led to similar results. **b** Measurements of [Ca^2+^] using recombinant aequorin-based Ca^2+^ sensors in the mitochondria upon agonist stimulation (100 μM Histamine) (*N* = 10). The traces are representative of ten independent experiments that led to similar results. **c** Flag and beta-Actin representative immunoblot of proteins extracted from SEPN1 KO HeLa cells transfected with SEPN1 wt-Flag, SEPN1 CC-Flag, and SEPN1 SS-Flag.

Since ER-to-mitochondria Ca^2+^ transfer occurs at the MAMs [40] and low mitochondrial calcium affects OXPHOS [41], we explored calcium levels in the ER lumen and mitochondria of WT and SEPN1-deficient cells, using ER- and mitochondria-localized aequorin Ca^2+^ sensors. Consistently with impaired SERCA activity [14, 16] and with previous studies in SEPN1-RM patient myotubes [12], SEPN1-deficient cells had less ER Ca^2+^ (Fig. 6a) and transferred less Ca^2+^ to mitochondria (Fig. 6b). Reintroduction of SEPN1 (with the native selenocysteine) improved calcium accumulation in the ER and calcium transfer to the mitochondria, contrary to two SEPN1 mutants [in which either the selenocysteine was mutated into the similar amino acid cysteine (SEPN1 CC), or the couple of redox amino acids CU were mutated in two serines (SEPN1 SS)] (Fig. 6a, c).

These ex vivo and in vitro data showed that SEPN1 is necessary for normal ER-mitochondria apposition at the MAM and for regulation of ER-mitochondria Ca^2+^ dynamics. Thus, the greater ER-mitochondria distance observed in SEPN1 KO models, together with altered ER Ca^2+^ homeostasis and Ψm, results in a drastic reduction of mitochondrial Ca^2+^ uptake, which in turn might affect OXPHOS and cell bioenergetics.

### SEPN1-RM patients have an age-related metabolic/bioenergetic phenotype and defective SR-mitochondrial contacts

To determine whether the pathomechanism identified in SEPN1-RM cellular and animal models is relevant to human disease, we investigated in-depth the bioenergetics/body composition phenotype in SEPN1-RM patients and its evolution with age.

**Fig. 7:**
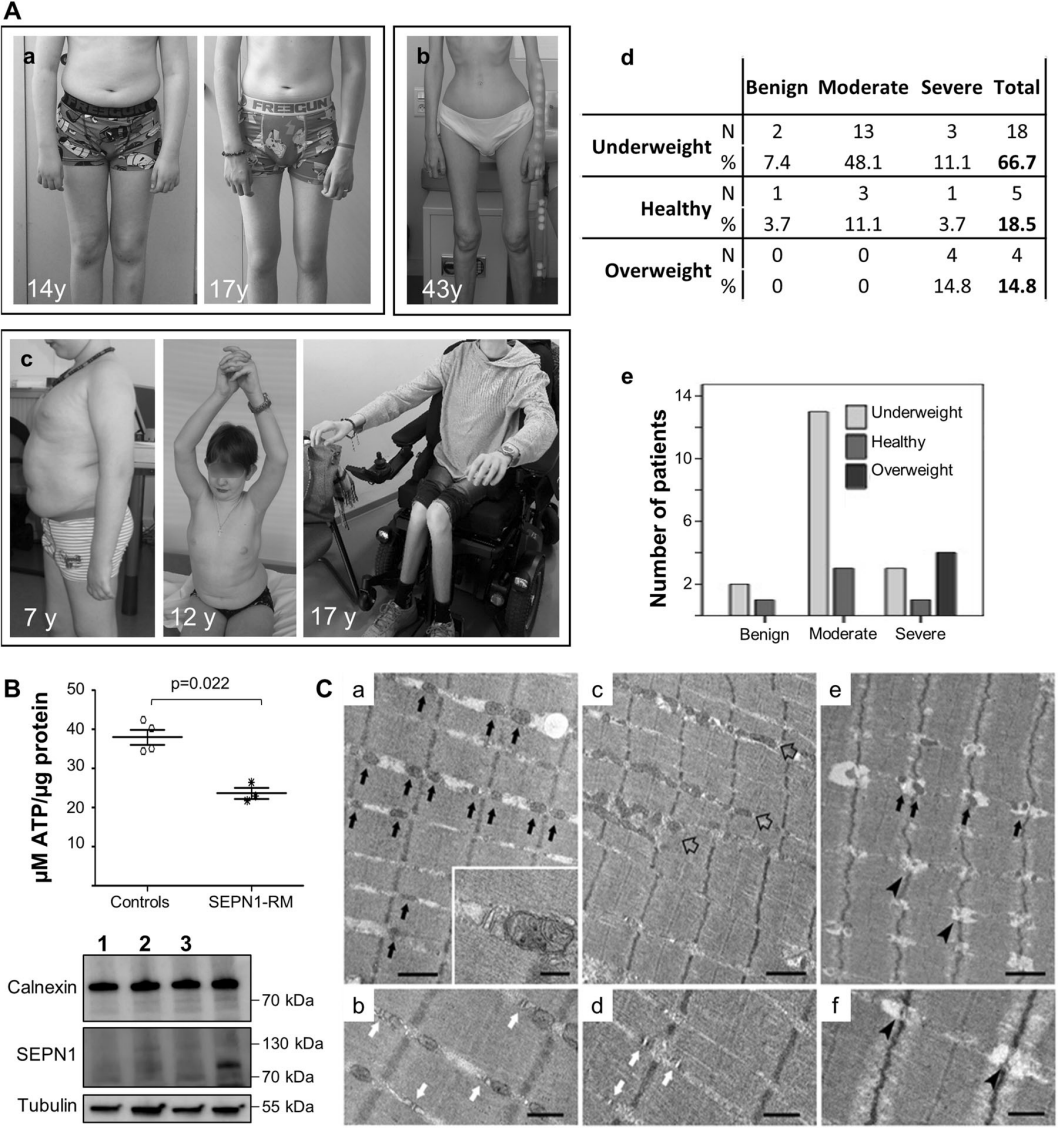
Abnormalities in body composition, ATP levels, and SR-mitochondrial contacts in patients confirm defective bioenergetics as a novel pathomechanism in SEPN1-RM. **A** Two typical SEPN1-RM patients showing loss of subcutaneous fat between the ages of 14 and 17 years (Patient a) and extremely reduced body mass with preserved ambulation in adulthood (Patient b). A small group of patients was in the overweight/obesity range, showed abdominal fat accumulation and had severe muscle weakness leading to loss of ambulation and reduced upper limb antigravity movements in their early teens (Patient c, wheelchair-bound from age 13 years). Anthropometric analysis (**d**) confirmed a positive correlation (*p* = 0.021) between BMI and disease severity (**e**). Most underweight patients had moderate disease, only a few of them having mild or more severe forms, while all the overweight cases were severe. **B** Reduced ATP content in primary fibroblasts from three patients homozygous or compound heterozygous for nonsense mutations leading to a severe reduction of SEPN1 protein (Western blot lanes 1–3; the last lane serves as a control to show SEPN1 expression), compared with four different age- and passage-paired controls. ATP was reduced in cells from both overweight/severe Patient 1 and underweight/moderated Patients 2–3. **C** Representative electron microscopy images from control (a–b), and SEPN1-RM patient (c–f) muscle biopsies. In control muscle fibers, mitochondria are positioned at the I band and often form pairs (a, black arrows) on both sides of Z lines; CRUs have the classic triad structure and are frequently associated with mitochondria (inset in a and white arrows in b). In muscle fibers from patients, mitochondria are usually misplaced from their correct triadic position, forming clusters and/or longitudinal columns between the myofibrils (empty arrows in c). Relocation of mitochondria leaves areas completely free of mitochondria (lower-right corner in c). In these areas, triads may still be present (white arrows in d). Intermyofibrillar white spots lacking mitochondria and triads are often present within apparently normal myofibrils (e, f, arrowheads). Scale Bars: a, c, e: 1 µm; b, d and f: 0.5 µm; inset: 0.2 µm.

Retrospective review of longitudinal follow-up biometric data available for a subgroup of 27 SEPN1-RM patients (15 females, 12 males, aged 7–46 years, mean 22.37 ± 10.55 years) included in a larger case series [20] confirmed that alterations of BMI are very frequent and thus part of the clinical phenotype of this disease. The mean BMI for the 27 patients was 16.97 ± 4.86, in the underweight range (normal = 18.5–25). Consistently with age-related loss of body weight in the murine SEPN1-RM model, a visible loss of subcutaneous fat was often observed around puberty in patients, leading to a cachexia phenotype (Fig. 7A, a–b). Moreover, the prepuberal mean BMI (ages 7–13, *N* = 5) was 20.46 ± 7.18 while the postpuberal/adult mean BMI dropped to 16.17 ± 4.12 (ages 14–42, *N* = 22), with numerous adult patients within the very severely underweight category (<15). When classified in three categories (underweight, normal weight, and overweight), two-thirds (66.67%) of the patients were underweight (<4th percentile), and only five patients (18.5%) were within the normal range. Remarkably, the remaining four patients (14.8%), whose global phenotype is detailed in [20], were overweight children (>85th percentile) and showed abdominal fat accumulation; two of them were above the 98th percentile, in the obesity range (Fig. 7A, c-d). To investigate whether body composition abnormalities, like muscle weakness, are a phenotypical manifestation of the underlying pathophysiological defect rather than an unrelated finding, we classified the patients in three groups (mild, moderate, and severe) according to the severity of muscle weakness and respiratory failure. Interestingly, we confirmed a significant correlation between body weight and disease severity (*p* = 0.021) (Fig. 7A, e). Most underweight patients had a moderate form of disease with preserved ambulation in adulthood (Fig. 7A, b). In contrast, all the patients who were overweight in childhood had severe weakness of both weight-bearing and non-weight-bearing muscles, requiring assisted ventilation early in childhood and leading to gait loss by their early teens (Fig. 7A, c).

Consistently, primary skin fibroblasts from three unrelated overweight/severe (*N* = 1) or underweight/moderate (*N* = 2) patients with *SEPN1* mutations leading to the absence of detectable SEPN1 protein showed 37.7% reduction of ATP content compared with age- and passage-paired controls (Fig. 7B). In agreement with the SEPN1-devoid models, this revealed that SEPN1 loss is associated with severe systemic bioenergetics dysfunction in patients, both in vivo and ex vivo.

In addition, we used TEM (transmission electron microscopy) to analyze the reciprocal association between the SR/calcium release units (CRUs) and mitochondria in muscle biopsies from three SEPN1-RM patients (Fig. 7C). In normal muscles, mitochondria are localized at the sarcomere I band level, on both sides of a triadic junction or CRU (Fig. 7C, a, b). In SEPN1-RM muscles, mitochondria were frequently misplaced, forming clusters and/or longitudinal rows (Fig. 7C, c–f). This mitochondria relocation left some fiber areas virtually without mitochondria. The close spatial correspondence between mitochondrial clusters and mitochondria-free areas and the frequent intermyofibrillar “white spots” in the I band regions (Fig. 7C, e, f) suggested that mitochondrial clusters are mostly the result of mitochondria moving away from their triadic intermyofibrillar location. In addition, SEPN1-devoid muscles had frequent regions in which CRUs were not coupled with mitochondria (Fig. 7C, d), while in others CRU and mitochondria disappeared (Fig. 7C, f). In the latter, myofibrils were often tightly packed together.

Quantification studies showed that, while both CRUs and mitochondria frequency were significantly reduced in SEPN1-RM patient muscles compared with controls, the relative fiber volume occupied by mitochondria was globally unchanged, suggesting dislocation of the mitochondria from their correct triadic position. This was strongly supported by the significantly increased aberrant localization of mitochondria at the A band (Table 1, Column D). Finally, the frequency of mitochondrion-CRU contacts per 100 μm^2^ (Table 1, Column E) was drastically reduced (down to 0) in fibers from SEPN1-RM patients.

**Table 1:** Quantitative analyses of CRUs and mitochondria frequency, mitochondria intracellular disposition, and CRUs-mitochondria association.

	A	B	C	D	E
	Number of CRUs/100 μm^2^	Number of mitochondria/100 μm^2^	Mitochondrial volume/total fiber volume %	Number of mitochondria at the A band/100 μm^2^	Number of CRU-mitochondria pairs/100 μm^2^
Control	12 ± 4	51 ± 11	6.8 ± 1.8	1 ± 1	7 ± 1
SEPN1-RM1	10 ± 6	24 ± 18*	6.6 ± 3.0	7 ± 7*	1 ± 1
SEPN1-RM2	1 ± 1*	16 ± 11*	5.9 ± 2.1	12 ± 9*	0 ± 0
SEPN1-RM3	1 ± 1*	12 ± 4*	3.1 ± 1.4*	12 ± 11*	0 ± 0

Data are shown as mean ± SD.

*p < 0.05 for Student’s *t* test.

These data suggest that SEPN1 loss in human muscle leads to the misplacement of mitochondria from the CRU, which might contribute to the reduced mitochondrial Ca^2+^ uptake and thus to OXPHOS and cell bioenergetic defects.

## Discussion

SEPN1-RM has brought to light the critical role of the putative reductase Selenoprotein N in skeletal muscle pathophysiology. Therefore, this rare disease represents a unique monogenic model for investigating defects in antioxidant defense and calcium handling in muscle physiology [12, 16]. It also represents a medical challenge given its peculiar phenotypical presentation, so far largely unexplained, and the absence of any validated treatments. Defining the pathomechanism of SEPN1-RM is a critical step towards developing biomarkers and pharmacological interventions, but also to understand the complex interactions between redox homeostasis and calcium handling and how they determine muscle mass, function, and myofiber architecture.

To meet metabolic demand during contraction and relaxation, skeletal muscle fibers require large amounts of ATP, mostly supplied by mitochondria through the complexes of the respiratory chain and OXPHOS system [42]. Contacts between the ER and mitochondria are functionally relevant for the transfer of Ca^2+^ and consequently for ATP generation [43, 44]. In murine skeletal muscles, ER and SR are closely associated to mitochondria [45] and about a quarter of the outer surface of mitochondria is close to the CRUs of the junctional SR, supporting the concept of high [Ca^2+^] microdomains which allow efficient Ca^2+^ uptake by mitochondria [46]. In turn, Ca^2+^ activates key mitochondrial metabolic enzymes, including those in complexes I, III, IV, and V of the electron transport chain. Mitochondrial calcium is therefore an important regulator of ATP production [47].

We show here that SEPN1-RM is a systemic bioenergetic disease, with defective energy production in muscle but also in extramuscular cells such as skin fibroblasts. Furthermore, age-related body mass abnormalities, involving cachexia and reduced muscle mass but also lipodystrophy-like adipose tissue accumulation, are part of the disease phenotype and correlate with the severity of muscle weakness. We also show that SEPN1 is enriched at the MAMs, and that its absence triggers ER stress, alters the ER/SR-mitochondria distance, ER Ca^2+^ levels, as well as mitochondrial membrane potential, thus impinging on the whole mitochondrial Ca^2+^ uptake and reducing ATP production. These findings help to explain the metabolic/systemic phenotype mentioned above, as well as the insulin resistance previously reported in SEPN1-RM patients [4, 13, 20]. They also help understanding the striking discordance in SEPN1-RM patients between the severe weakness of axial muscles (such as paravertebral and diaphragm), which are highly dependent on oxidative metabolism, and the relative preservation of extraocular or limb muscles such as quadriceps, with higher glycolytic metabolism. Importantly, our findings and particularly defective ATP production provide a mechanistic explanation to the muscular and diaphragmatic fatigue which plays a key role in the life-threatening respiratory failure typical of SEPN1-RM [18], and has a major impact on patient’s functional performances and quality of life [13]. The positive correlation between abdominal adiposity and severity of muscle weakness (including non-weight-bearing muscles) contrasts with the overall weight loss and decreased LBM observed nonspecifically in many severe congenital myopathies. This correlation could be a consequence of extrinsic dietary factors, high-fat diet being a modifier of ER stress recently associated with SEPN1 defects [15]. A putative alternative explanation could be a particularly serious intrinsic mitochondrial dysfunction in the overweight/severe patients, preventing use of fatty acids as a mitochondrial substrate and leading to their secondary accumulation in the subcutaneous tissue and to a major bioenergetic defect (hence weakness) in muscles. A comparative study of muscle-specific versus constitutive SEPN1 KO would be theoretically useful to investigate potential underlying whole-body adaptive mechanisms, but the subtlety of the murine phenotype even upon constitutive SEPN1 deficiency hinders this approach. Further studies based on the new concepts reported here, including muscle NMR spectroscopy studies in patients, should help to clarify this point.

The origin of the small focal areas of mitochondria depletion and sarcomere disorganization in muscle fibers (minicores), which characterize SEPN1-RM but also other forms of congenital myopathy [48], remained unknown. SEPN1 localization at the MAM and loss of SR/mitochondrial contacts in muscles from patients devoid of full-length SEPN1 suggest that a so far unreported tethering role of this protein underlies the generation of minicores. These findings corroborate previous observations showing that abnormalities in mitochondria and focal Ca^2+^ handling precede sarcomere disruption in core lesions associated with defects of the calcium release channel RyR1 [49]. Interestingly, altered ER/SR-mitochondria distance was also reported in RyR1^I4895T^ heterozygous knock-in mice, expressing a mutation orthologous to I4898T in human RyR1 which causes a congenital core myopathy (central core disease) [50, 51]. Moreover, similar alterations of ER/SR-mitochondria contacts have been identified in a few other models of neuromuscular diseases associated with ER stress as well as in in aging muscles [52, 53]. Thus, a mislocalization of ER/SR from mitochondria may be an overlooked defect not only in myopathies due to defects in calcium-handling proteins but also in aging, often associated with muscle fatigue/exercise intolerance and body composition changes (cachexia or sarcopenia).

In conclusion, Selenoprotein N is a pivotal actor in a so far unrecognized interplay between mitochondrial bioenergetics, ER/SR and redox homeostasis in skeletal muscle. Further studies are in progress to determine whether this is due to a primary role of SEPN1 as an ER-mitochondria tethering protein or to less direct mechanisms. In any case, our results pave the way to the identification of biomarkers and therapeutic drugs for SEPN1-RM. They also suggest that SEPN1-RM might represent a useful pathophysiological and therapy-development model for other inherited muscle disorders, but also for prevalent conditions in which body mass composition and/or muscle ER/SR-mitochondria cross-talk are impaired.

## Supplementary information

Supplementary Figure 1

Legend to Supplementary Figure 1
