# Supplementary figures and images for: Defective endoplasmic reticulum-mitochondria contacts and bioenergetics in SEPN1-related myopathy
Source: Cell Death Differ. 2020 Jul 13;28(1):123–38. doi: 10.1038/s41418-020-0587-z (PMC7853070; doi:10.1038/s41418-020-0587-z)

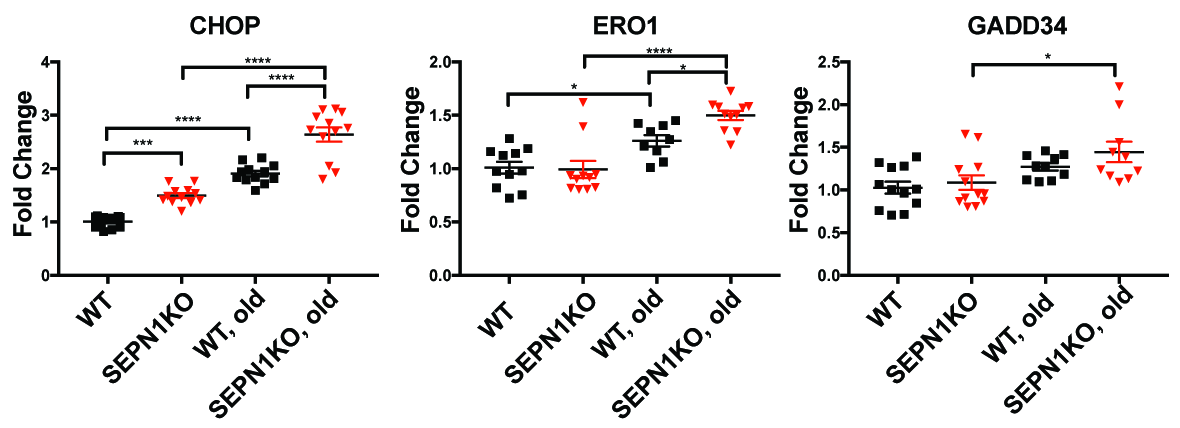

Supplement: Supplementary file 1 — Supplementary Figure 1 [file 41418_2020_587_MOESM1_ESM.tif]
