# Supplementary material for: Defective endoplasmic reticulum-mitochondria contacts and bioenergetics in SEPN1-related myopathy
Source: Cell Death Differ. 2020 Jul 13;28(1):123–38. doi: 10.1038/s41418-020-0587-z (PMC7853070; doi:10.1038/s41418-020-0587-z)
Supplement: Supplementary file 2 — Legend to Supplementary Figure 1 [file 41418_2020_587_MOESM2_ESM.docx]

**Fig. SUP 1 ER stress/maladaptive ER stress response in SEPN1 KO muscles**

Semi-quantitative, real-time RT-PCR analysis of ER stress response markers in mRNA prepared from diaphragms of young (5-month-old) and old (18-month-old) WT and SEPN1 KO mice. Data are shown as mean ± SEM (One-way Anova, Sidak test).
